# Occurrence of Virulence Genes Associated with Human Pathogenic Vibrios Isolated from Two Commercial Dusky Kob (*Argyrosmus japonicus*) Farms and Kareiga Estuary in the Eastern Cape Province, South Africa

**DOI:** 10.3390/ijerph14101111

**Published:** 2017-09-25

**Authors:** Justine Fri, Roland Ndip Ndip, Henry Akum Njom, Anna Maria Clarke

**Affiliations:** 1 Microbial Pathogenicity and Molecular Epidemiology Research Group (MPMERG), Department of Biochemistry and Microbiology, University of Fort Hare, Alice 5700, South Africa; ndip3@yahoo.com (R.N.N.); hnjom@ufh.ac.za (H.A.N.); aclarke@ufh.ac.za (A.M.C.); 2 Department Microbiology and Parasitology, University of Buea, P.O. Box 63, Buea, Cameroon

**Keywords:** marine fish, human pathogenic vibrios, virulence, human health

## Abstract

*Background*: Seafood-borne *Vibrio* infections, often linked to contaminated seafood and water, are of increasing global public health concern. The aim of this study was to evaluate the prevalence of human pathogenic vibrios and their associated virulence genes isolated from fish and water samples from 2 commercial dusky kob farms and Kareiga estuary, South Africa. *Methods*: A total of 200 samples including dusky kob fish (*n* = 120) and seawater (*n* = 80) were subjected to *Vibrio* screening on thiosulfate-citrate-bile salts-sucrose agar (TCBS). Presumptive isolates were confirmed and delineated to *V. cholerae*, *V. parahaemolyticus*, *V. vulnificus*, and *V. fluvialis* by PCR. Various pathogenic gene markers were screened: *V. parahaemolyticus* (*trh* and *tdh*), *V. vulnificus* (*vcgE* and *vcgC)* and *V. fluvialis (stn, vfh,* *hupO*, *vfpA*). Restriction Fragment Length Polymorphism (RFLP) of the *vvh*A gene of *V. vulnificus* strains was performed to determine the associated biotypes. *Results*: Total *Vibrio* prevalence was 59.4% (606/1020) of which *V. fluvialis* was the most predominant 193 (31.85%), followed by *Vibrio vulnificus* 74 (12.21%) and *V. parahaemolyticus* 33 (5.45%). No *V. cholerae* strain was detected. One of the *V. parahaemolyticus* strains possessed the *trh* gene 7 (9.46%) while most (91.9%; 68/74) *V. vulnificus* isolates were of the E-type genotype. *V. fluvialis* virulence genes detected were *stn* (13.5%), *hupO* (10.4%) and *vfpA* (1.0%). 12.16% (9/74) of *V. vulnificus* strains exhibited a biotype 3 RFLP pattern. *Conclusions*: This is the first report of potentially pathogenic vibrios from healthy marine fish in the study area, and therefore a public health concern.

## 1. Introduction

In recent years, there have been concerns about the microbiological safety of fish and other seafood due to increased outbreaks of seafood-borne pathogens. Members of the genus *Vibrio* are among the bacterial pathogens increasingly implicated in seafood-associated infections [1]. Although not all species of the genus are pathogenic to humans, incidences of human *Vibrio*-related illnesses over the last few years have been on the rise, particularly in developing countries, with *Vibrio cholerae*, *V. vulnificus*, *V*. *parahaemolyticus and V. fluvialis* being the most important [2,3]. Only toxigenic strains of O1 and O139 *V. cholerae* are associated with cholera illness; however, other strains belonging to non-O1/non-O139 have been associated with sporadic outbreaks of diarrhoea through the ingestion of contaminated seafood [4]. *V. parahaemolyticus* is one of the leading causes of seafood-associated diarrhoeal infection in many countries including the United States, Asian and other developing countries [5,6,7,8,9]. It is one of the frequent causative agents of gastroenteritis, caused by the consumption of raw, undercooked seafood. Although it is best known for causing gastroenteritis, cases of wound infections and septicaemia caused by *V. parahaemolyticus* have been reported [6,10]. *Vibrio vulnificus*, on the other hand, is a notorious pathogen in aquaculture and marine environments. It is second to *V. parahaemolyticus* as the most frequently encountered bacterial species that cause infection of food-borne origin and has received attention due to the high mortality rate of infection, making it a significant health risk [11]. Infection with this pathogen is invasive, and it causes either gastroenteritis or necrotizing wound infections, both which may lead to septicaemia [5]. Three *V. vulnificus* biotypes have been described. Biotype 1 is the most common worldwide, is primarily a human pathogen, and is responsible for numerous clinical cases of the disease [12]. Biotype 2 strains are primarily eel pathogens, which cause eel vibriosis or death and may be opportunistic for humans [13]. Biotype 3, is the most recently discovered, is known to be geographically restricted to Israel where it caused an outbreak of disease in fish farmers and consumers of Tilipia fish [12,14]. *Vibrio fluvialis* has been termed an “emerging” pathogen, due to the increased numbers of cholera-like diarrheal outbreaks and sporadic extra-intestinal cases attributed to it [15]. In the last decade, it has been reported in a variety of sources including aquaculture [16], final effluents of waste water treatment plants [17], fish and other seafood [18].

Human pathogenic vibrios produce a wide array of virulence factors which contribute to mild to fatal illnesses [2]. Largely, *V. cholerae’s* ability to cause disease is dependent on the production of two major virulence determinants, cholera toxin (CT) and the toxin-coregulated pilus (TCP). The CT is encoded by the *ctxA* and *ctxB* genes found on the intergrated prophage CTXϕ and it is responsible for the manifestation of diarrhoea with severe water and electrolyte loss [19]. The TCP on the other hand, encoded by *tcpA*, is required for *V. cholerae* colonisation of the small intestinal epithelium [19]. The expression of these genes is controlled by the *toxR* regulon, in response to in vivo stimuli [20]. The thermostable direct hemolysin (*tdh*), in particular, as well as the thermostable direct hemolysin-related gene (*trh*) are major contributors to the pathogenicity of *V. parahaemolyticus* [8,21], owing to its biological activities, including its cytotoxic and enterotoxic effects [10]. The virulence of *V. vulnificus* is mainly coded for by the virulence-correlated gene (*vcg*). Differences in virulence degrees have been found in two major genotypes, with the degree of virulence being related to the origin of the strain, with clinical strains displaying higher virulence than environmental isolates [22]. *V. fluvialis*, on the other hand is known to produce several potent toxins including the heat stable enterotoxin (*stn*), although their roles in pathogenesis are not well established [23]. However, as is the case with *V. cholerae*, the *toxR* plays an important role in the bile resistance of *V. fluvialis*, an initial phase in the establishment of the disease. Other virulence factors include the heme utilization protein gene (*hupO*), extracellular haemolysin gene (*vfh*), and the *V. fluvialis* protease gene (*vfp*) [24].

A few reports on pathogenic vibrios in South Africa have been made, mostly from final effluents of waste water treatment plants [25,26,27]. However, recent epidemiological data is lacking. There is also little attention being paid to the occurrence of these pathogens in seafood or fish of commercial interest, which could be major source of seafood infection. We therefore investigated the occurrence of human pathogenic vibrios in dusky kob fish as well as water samples from two commercial farms and the Kareiga Estuary and investigated the presence of virulent associated genes that may aid in pathogenicity, which presents a guide to the risk they may pose to the community.

## 2. Materials and Methods

### 2.1. Sampling

An ethical clearance for the study was obtained from the University of Fort Hare Research Ethics Committee (UREC) (CLA011SFRI01). Water and fish samples were harvested from two aquaculture dusky kob farms: Farm 1, with coordinates 13.0670° N, 59.5712° W, and Farm 2, with coordinates 32.9638° S, 27.8789° E, in the Eastern Cape Province and from the Kariega Estuary (33°41′ S, 24°44′ E). Samples from the wild were purchased directly from fishermen as they came ashore or captured by fishing experts from SAIAB (South African Association for Aquatic Biodiversity). Upon capture, all fish were inspected for signs of any disease or abnormality, of which none were evident. Therefore, only healthy juveniles of consumption size (body mass of ≥0.6 Kg), approximately ≥7 months old were included in the study. Sampling was spread between November 2014 and October 2015, spanning the four seasons of the year. A total of 120 dusky kob fish (100 from fish farms and 20 from the wild) and 80 water samples were included in the study. The fish were ice killed and placed immediately in sterile zip lock bags post-harvest. Water was aseptically collected in sterile 2 L bottles from fish tanks at harvest as well as from the Kariega estuary during each sampling. Fish and water samples were transported in cool chain to the laboratory and processed within 4 h of collection.

### 2.2. Bacteriological Analyses

The outer surfaces of the fish were rinsed with sterile distilled water prior to bacteriological analysis. The skin, gill and gut were aseptically excised with a scalpel and scissors and homogenised. A 1:10 broth dilution enrichment of homogenate into alkaline peptone water (Oxoid, Basingstoke, UK), pH 8.6, was carried out and this was incubated at 37 °C for 4–6 h, and further sub-cultured on thiosulfate-citrate-bile salts-sucrose (TCBS) agar (Oxoid, Basingstoke, UK). To enhance detection of *Vibrio* species from water samples, analysis was done according to American Public Health Association (APHA) [28], where 100 mL each of water samples were filtered through a 0.45-μm membrane, with the membrane inoculated on TCBS (Oxoid, Basingstoke, UK) as well as pre-enrichment of water samples in APW prior to culture on TCBS. Following 24–48 h incubation, 2–5 distinctive presumptive *Vibrio* colonies per TCBS plate were inoculated in brain heart infusion (BHI) agar (Oxoid, Basingstoke, UK) for purity and incubated at 37 °C for 24 h. Preliminary tests on presumptive isolates included gram staining, oxidase (using of oxidase test strips (MB0266A, MICROBACT™ Oxoid, Basingstoke, UK) and catalase tests.

### 2.3. Molecular Confirmation of Vibrio Species

Presumptive *Vibrio* isolates were subjected to a polymerase chain reaction (PCR) assay for molecular identification. DNA was isolated by the boiling method where pellets from 18–24 h broth cultures were suspended in 200 µL of sterile distilled water, and cells lysed by boiling (100 °C for 15 min) in a digital Accu dri-block (Lasec, Capetown, SA). Cell debris was removed by centrifugation at 13,000× *g* for 5 min and the supernatants were used directly as templates in PCR reactions or stored in 50 µL aliquots at −20 °C until use. Primers targeting the 16S rRNA gene variable region that spans between 700 bp and 1325 bp were used to confirm whether isolates belonged to the genus *Vibrio* [29]. Confirmed isolates were further delineated to four human pathogenic species: *V. cholerae*, *V. paraheaemolyticus*, *V. vulnificus and V. fluvialis. Vibrio cholerae*, *V. paraheaemolyticus* and *V. vulnificus* were detected by multiplex PCR using species-specific primers as reported earlier [30], while single PCRs were performed to detect *V. fluvialis* [23]. *Vibrio cholerae* was also detected by targeting the *ompW* gene [31]. The various primer sets used for PCR confirmation and species differentiation are shown on Table 1. *V. vulnifius* ATCC 27562, *V. parahaemolyticus* ATCC 17802 and *V. fluvialis* ATCC 33809, as well as extracted DNA from a confirmed O139 *V. cholerae* strain (obtained from the Microbiology laboratory of the Natural Resources and the Environment (NRE), Council for Scientific Research (CSIR)) were used as positive controls.

### 2.4. Detection of Virulence Genes

PCR confirmed *V. parahaemolyticus* isolates were screened for the presence of *tdh and trh* virulence genes using previously reported primers [32], while *V. vulnificus* virulence genes (*vcgE and vcgC*) were detected as previously described [33]. Four gene-specific primer sets (Table 2) were used in a touch-down multiplex PCR amplification of *V. fluvialis* virulence genes, heat stable enterotoxin (*stn*), heme utilization protein gene (*hupO*), extracellular haemolysin gene (*vfh*) and *V. fluvialis* protease gene (*vfp*) [24]. Table 2 indicates the target genes and primer sets used for detection of *Vibrio* virulence genes. All amplifications were performed in 25 µL reactions, each consisting of 12.5 µL of 2× master mix (Thermo scientific, Johannesburg, South Africa), 0.5 µL of each oligonucleotide (Inqaba Biotec, Pretoria, South Africa), and an appropriate volume of nuclease free water (Thermo scientific, Waltham, MA, USA). PCR products were separated by electrophoresis in 1–2% agarose gels containing 5 µg/mL ethidium bromide (Sigma-Aldrich, St. Louis, MO, USA) run at 100 V for 45 min and visualised under a UV transilluminator (Alliance 4.7 XD-79, Uvitec, Cambridge, UK).

### 2.5. Biotyping of V. Vulnificus

The restriction fragments of the *vvh*A gene obtained using *Kpn*I or *Pst*I can be used to differentiate biotype 3 from biotypes 1 and 2 [34]. The *vvh*A nucleotide sequence of biotype 3 strains contain restriction sites for these enzymes while those of biotypes 1 and 2 do not. Biotyping of the confirmed *V. vulnificus* strains was therefore carried out as earlier reported [34]; the *V. vulnificus* cytotoxin-hemolysin gene (*vvhA*) was amplified using the primer pair Fw 5′ CAGCTCCAGCCGTTAACCGAACCACCCGC-3′ and Rv 5′-TTCCAACTTCAAACCGAACTATGAC-3′. Amplicons were resolved on 1.5% agarose gel followed by restriction fragment length polymorphism (RFLP). The two restriction enzymes, (*Kpn*I and *Pst*I), were used for restriction digest following the manufacturer’s instructions (New England Biolabs, Ipswich, MA, USA). The restricted digests were separated by electrophoresis in 2% agarose gels and visualised in a UV transilluminator (Alliance 4.7 XD-79, Uvitec, Cambridge, UK).

## 3. Results

### 3.1. Prevalence of Vibrio Species

**Figure 1:**
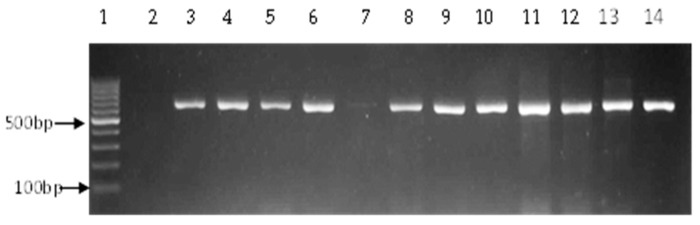
Representative gel showing the 663 bp 16S rRNA PCR amplified gene for confirmation of *Vibrio* isolates. Lane 1: molecular weight marker (100 bp), lane 2: negative control, lane 3: positive control (ATCC 17802), lanes 4–6 and 8–14: positive isolates, lane 7: negative isolate.

**Figure 2:**
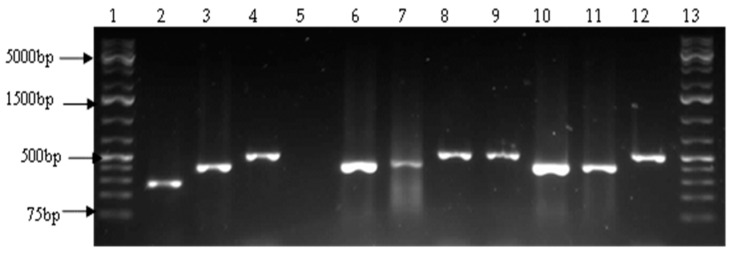
A representative agarose gel showing the 275 bp, 366 bp and 503 bp PCR products of *V. cholerae toxR*, *V. vulnificus vvhA* and *V. parahaemolyticus toxR* genes respectively. Lanes 1 and 13: molecular weight markers (1 kb plus), Lane 2: *V. cholerae* positive control (O139 positive DNA from CSIR, SA), lane 3: *V. vulnificus* positive control (ATCC 27562), lane 4: *V. parahaemolyticus* positive control (ATCC 17802), Lane 5: negative control, Lanes 6, 7, 10 and 11: *V. vulnificus* positive isolates, and lanes 8, 9 and 12: *V. parahaemolyticus* positive isolates.

**Figure 3:**
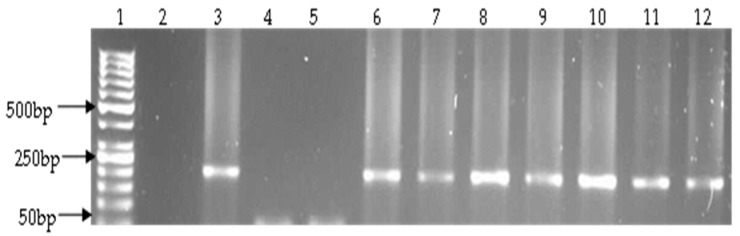
Representative gel showing the 217 bp PCR amplified *V. fluvialis toxR* gene. Lane 1: molecular weight marker (50 bp), lane 2: negative control, lane 3: positive control (ATCC 33809), lanes 4 and 5: negative isolates, lanes 6–12: positive isolates.

A total of 1020 presumptive isolates were obtained by culture, of which 606 (59.4%) were positive by PCR as belonging to the genus *Vibrio* (Figure 1). *Vibrio fluvialis* was the most predominant, 193 (31.85%), followed by *V. vulnificus*, 74 (12.21%) and *V. parahaemolyticus* 33 (5.45%) (Table 3, Figure 2 and Figure 3). No *V. cholerae* was detected. The rest of the isolates, 306 (50.5%) were assumed to belong to other species of the genus not considered in this study.

### 3.2. Prevalence of Vibrio Virulence Genes

**Figure 4:**
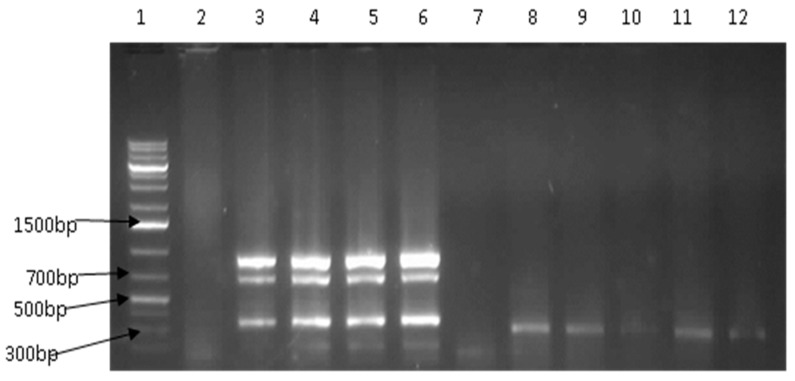
Representative gel of multiplex PCR detected *V. fluvialis* virulence genes. Lane 1: molecular weight marker (1 kb plus), lane 2: negative control, lane 3: positive control, lanes 4–6, 8, 9, 11 and 12: positive for *stn* (375 bp), lanes 4–6: positive for *vfh* (800 bp), lanes 4–6: positive for *hupO* and lanes 7 and 10: negative isolates.

Most (97.0%; 32/33) *V. parahemolyticus* species were negative for *tdh* gene, while one wild fish isolate was positive for *trh* (3.0%; 1/33). Genetic variations in the virulence-correlated gene (*vcg*) serve as a primary feature to distinguish clinical (C-) genotypes from environmental (E-) genotypes. Of the 74 confirmed *V. vulnificus* isolates, 6 (8.1%) had the *vcgC*, while 68 possessed *vcgE* (91.9%). *V. fluvialis* was screened for the presence of four virulence genes, *vfh*, *hupO*, *stn*, and *vfp*A. A total of 35 isolates possessed one to three of these genes, with *stn* being the most prevalent 13.5% (26/193), followed by *hupO*, 10.4% (20/193) and *vfh* 6.2% (12/193) (Table 4, Figure 4). None of the isolates possessed the *vfpA* virulence gene.

Of the 35 *V. fluvialis* strains that tested positive for virulence genes, three strains were *vfh-hupO-stn* positive, eight strains were *vfh*-*hupO* positive, eight were *hupO-stn* positive and one strain was *vfh-stn* positive. The rest were either positive for *stn* (1/35) or *hupO* (14/35) only (Table 5).

**Figure 5:**
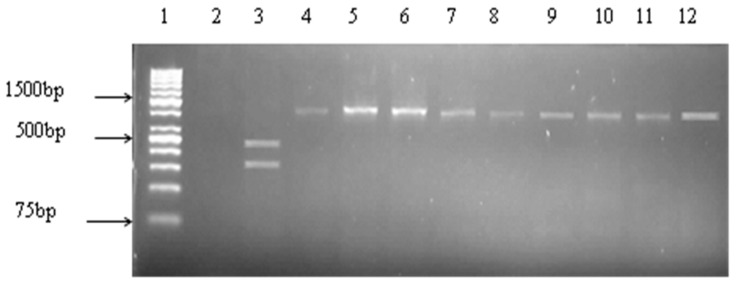
Representative gel of PCR RFLP of *Vibrio vulnificus vvhA* gene. Lane 1: molecular weight marker (1 kb plus), lane 2: negative control, lane 3: *V. vulnificus* biotype 3, lanes 4–12, *V. vulnificus* biotypes 1 and 2.

For all *V. vulnificus* strains, the *vvh*A restriction fragment(s) obtained when digested with *Kpn*I were similar to those obtained when digested with *Pst*I. Restriction sites for both enzymes were present in nine (12.16%) of the nucleotide sequences of the *vvh*A gene revealing biotype 3 with no restriction sites for the majority (87.84%, 65/74), indicating biotypes 1 and 2 (Figure 5).

## 4. Discussion

Marine filter feeders are highly susceptible to surface or tissue contamination of marine origin, which may include potential human pathogens. Other marine foods are not exempt from bacterial contamination either. Members of the genus *Vibrio* are ubiquitous in marine and estuarine waters and therefore the high total prevalence (59.4%) recorded in our study was not surprising. What concerned us was the presence of virulence traits among potentially pathogenic vibrios.

Three (*V. parahaemolyticus, V. fluvialis,* and *V. vulnificus*) of the four human pathogens considered in this study were detected from the fish and water samples, with some strains harboring one or more virulence determinants. This is similar to an earlier study [35] reporting a number of *Vibrio* species which are indigenous aquatic bacteria that contaminate fish, including *V. cholerae*, *V. parahaemolyticus*, and *V. vulnificus,* mostly present in warm (>15 °C) saline waters, and are implicated in many food-borne illnesses. The most frequently isolated non-cholera-causing human pathogenic *Vibrio* species was *V. fluvialis*, 31.85% (193/606). This result is similar to others previously reported where *V. fluvialis* was the most frequently occurring species isolated from fish ponds [36], as well as in a variety of seafood [37]. The high frequency of occurrence of this pathogen in marine reservoirs could point towards why an increasing number of cases in the last decade have been reported terming it “emerging”. First reported in 1975 in a patient with diarrhoea in Bahrain [38], *V. fluvialis* has since become distributed worldwide, and is associated with numerous reports of food poisoning [39,40], as well as gastroenteritis with diarrheal illness connected with the consumption of raw or improperly cooked seafood [41,42]. We also detected *V. vulnificus* (12.21%) and *V. parahaemolyticus* (5.45%) although at lower frequencies than those recorded in waste water treatment facilities [25,26,27]. The presence of this pathogen in marine fish and aquaculture could be a source of infection to humans, especially to the immunocompromised. Earlier reports in Israel in 1996 and 1997 [12], implicated *V. vulnificus* in an outbreak of invasive infection in handlers of whole fresh fish purchased from fish ponds. *V. vulnificus* was also the source of necrotizing fasciitis that led to the amputation of the lower limb of a 17 year old boy after minor trauma during exposure to a contaminated fish tank [43]. Although cases of *V. vulnificus* infection may be generally low, it has received attention due to high case fatalities related to consumption of contaminated seafood [44,45].

Bacterial pathogenicity depends on the production of virulence factors, with virulence genes acting as major orchestrators. The dominant virulence factors of *V. parahaemolyticus* with regard to its pathogenicity are *tdh* and *trh* [10]. All *V. parahemolyticus* strains detected in this study were negative for the *tdh* gene, while *trh* was detected in one isolate (1.4% (1/33). These results are, however, not surprising, as previous reports have indicated the presence of these virulent signatures in only low numbers (1–7%) in environmental samples [46]. Similarly, *Vibrio* isolates recovered from West Sumatran rivers and shrimp farms in Sri Lanka were void of these virulence genes [47,48]. A low prevalence (0% *tdh* and 4.2% *trh*) was also recorded from 96 seafood and water samples in Turkey [8]. Although clinical cases of *V. parahaemolyticus* are associated with increased detection of these genes, the continuous report of pathogenic *V. parahaemolyticus* in the last few years and the fact that it is the most commonly encountered *Vibrio* species implicated in seafood infection is an indication that other virulence factors may exist [49,50,51]. Mahoney et al. [52] reported environmental isolates of *V. parahaemolyticus* lacking the *tdh* and *trh* but producing putative virulence factors such as extracellular proteases, biofilm and siderophores. Recently, Raghunath reported finding environmental isolates of *V. parahaemolyticus* lacking the *tdh* and *trh* genes but highly cytotoxic to human gastrointestinal cells [53]. Some *Vibrio fluvialis* strains in this study were found to possess three of the four targeted virulent genes, *stn* 13.5%, *hupO* 10.4% and *vfh* 6.2%. Although prevalence of these genes may be low, it should not be ignored as there are increased reports of this pathogen in the last decade [36,37,40]. Moreover, some of these virulent genes may be transferred to similar or other bacterial species via horizontal gene transfer or mobile genetic elements, leading to virulent human infections. The exotoxin-hemolysin *vvh*A virulence protein produced by *V. vulnificus* facilitates the release of iron from haemoglobin. It also contributes to the bacteria’s virulence by producing cytotoxic effects [54]. The virulence correlated gene *vcg*, however, has been one of the major virulence factors of *V. vulnificus* and can be used to distinguish potentially virulent from avirulent strains. The *vcgC* gene is linked mainly with clinical isolates while the *vcgE* is linked mostly with environmental isolates [55]. In this study, over 90% of *V. vulnificus* isolates were of the *vcgE* genotype, an indication of less virulent strains. This result is similar to that of a study recording a significantly higher percent of the E-genotype of *V. vulnificus* isolates recovered from oysters [56]. Although such a result is expected from environmental samples, it is, however, contrary to a report where nearly an equal percent of *vcgE* (47%) and *vcgC* (53%) were detected from oyster isolates [57] and water areas surrounding oyster harvest [56]. Continuous monitoring of seafood and fish products remains a necessity as infection with *V. vulnificus*, although rare, is usually fatal, particularly in immunocompromised persons.

Restriction digest of *V. vulnificus vvh*A gene revealed that most of the isolates belonged to biotypes 1 and 2, while a small percentage (12.16%), belonged to the biotype 3. Biotype 1 is primarily a human pathogen, and the leading cause of seafood-related deaths due to primary septicaemia [54], while biotype 2 mainly causes eel vibriosis [13]. However, Amaro and Biosca [58], reported a clinical *V. vulnificus* strain isolated from a human wound which belonged to biotype 2, suggesting that biotype 2 pathogenic for eels are also opportunistic for humans. In addition, some biotype 2 strains isolated from human samples could not be definitively linked to eel exposure or manipulation despite earlier reports to the contrary [13]. Seawater and fish species other than eels have been suggested, therefore to be putative reservoirs of biotype 2 [59]. Results from our study indicate that dusky kob and its surrounding waters are likely to be reservoirs for *V. vulnificus*, which could either cause infection or be opportunistic for humans particularly in persons with underlying disease. The first reported case of *V. vulnificus* biotype 3 determined to be the cause of severe soft tissue infections, and associated with exposure to contaminated fish, (especially tilapia or common carp), was described in 1996–1997 [34]. This biotype is thought to be geographically restricted to Israel. Our study is the first report of *V. vulnificus* biotype 3 isolated from a marine source in South Africa.

## 5. Conclusions

To the best of our knowledge, our findings represent the first report on the prevalence of human pathogenic *Vibrio* species with some virulent signatures isolated from healthy marine fish in South Africa. Although no *Vibrio cholerae* was detected, the occurrence of other human pathogenic vibrios, including *V. parahaemolyticus, V. fluvialis* and *V. vulnificus*, (all of which can cause sporadic and epidemic gastroenteritis as well as other serious diseases), is of public concern. Detection of *V. vulnificus* biotype 3 indicates its circulation in the region. Even though transmission of fish pathogens to humans may be relatively low, it is a health risk that needs to be considered by fish farmers, fishermen and others who handle or consume fish products, particularly in conjunction with the increased rate of seafood-associated *Vibrio* infections. Consumption of properly cooked fish is recommended, while frequent epidemiological studies could be carried out to better understand the associated health risk and implement control strategies for reduction of possible human infections.

## Figures and Tables

**Table 1:** Primer sequences and expected amplicon size of PCR amplified gene targets for *Vibrio* species.

Target Species	Target Gene	Primer Sequence (5′–3′)	Amplicon Size (bp)	Cycling Conditions	References
*Vibrio*	16S *rRNA*	CGGTGAAATGCGTAGAGAT TTACTAGCGATTCCGAGTTC	663	Initial denaturation at 94 °C for 5 min, followed by 35 cycles at 94 °C for 30 s, 52 °C for 30 s and 72 °C for 60 s. Final extension was at 72 °C for 10 min.	[29]
*V. parahaemolyticus*	*toxR*	TGTACTGTTGAACGCCTAA CACGTTCTCATACGAGTG	503	Initial denaturation at 94 °C for 5 min, followed by 35 cycles of 94 °C for 30 s, 55 °C for 30 s and 72 °C for 30 s. A final extension step at 72 °C for 10 min.	[30]
*V. vulnificus*	*vvhA*	ACTCAACTATCGTGCACG ACACTGTTCGACTGTGAG	366		
*V. cholerae*	*toxR*	GAAGCTGCTCATGACATC AAGATCAGGGTGGTTATTC	275		
	*OmpW*	CACCAAGAAGGTGACTTTATTGTG GGTTTGTCGAATTAGCTTCACC	304	Initial denaturation at 94 °C for 10 min, followed by 30 cycles at 94 °C for 60 s, 59 °C for 60 s and 72 °C for 2 min. Final elongation step at 72 °C for 10 min.	[31]
*V. fluvialis*	*toxR*	GGATACGGCACTTGAGTAAGACTC GACCAGGGCTTTGAGGTGGACGAC	217	Initial denaturation step at 94 °C for 5 min, followed by 30 cycles at 94 °C for 30 s, 57 °C for 60 s and 72 °C for 90 s. A final extension step at 72 °C for 7 min.	[23]

**Table 2:** Primer sets used in the detection of *Vibrio* virulence genes.

Primer	Sequence (5′–3′)	Product Size (bp)	Cycling Conditions	References
*tdh*F *tdh*R	GGTCTAAATGGCTGACATC CCACTACCACTCTCATATGC	199	Initial denaturation at 96 °C for 5 min, followed by 35 cycles of 94 °C for 60 s, 55 °C for 60 s and 72 °C for 60 s. Final extension at 72 °C for 7 min.	[32]
*trh*F *trh*R	CATTTCCGCTCTCATATGC GGCTCAAAATGGTTAAGCG	250		
*vcgC*P1 *vcg*P3	AGCTGCCGATAGCGATCT CGCTTAGGATGATCGGTG	278	Initial denaturation at 94 °C for 5 min, followed by 35 cycles of 94 °C for 40 s, 56°C for 40 s and 72 °C for 60 s. Final extension at 72 °C for 7 min.	[33]
*vcgE*P2 *vcg*P3	CTCAATTGACAATGATCT CGCTTAGGATGATCGGTG	278	Initial denaturation at 94 °C for 5 min, followed by 35 cycles of 94 °C for 40 s, 49 °C for 40 s and 72 °C for 60 s. Final extension at 72 °C for 7 min.	[33]
*vfh*-F *vfh*-R	GCGCGTCAGTGGTGGTGAAG TCGGTCGAACCGCTCTCGCTT	800	Initial denaturation at 93 °C for 15 min, followed by 35 cycles at 92 °C for 40 s, 50–62 °C for 60 s and 72 °C for 90 s. Final elongation step at 72 °C for 7 min.	[24]
*hupO*-F *hupO*-R	ATTACGCACAACGAGTCGAAC ATTGAGATGGT AAACAGCGCC	600		
*vfpA*-F *vfpA*-R	TACAACGTCAAGTTAAAGGC GTAGGCGCTGTAGCCTTTCA	1790		
*stn*-F *stn*-R	GGTGCAACATAATAAACAGTCAACAA TAGTGGTATGCGTTGCCAGC	375		

**Table 3:** Distribution and frequency of occurrence of PCR confirmed *Vibrio* strains isolated from water and fish samples.

Sampling Site	Source (*n* = No. of *Vibrio* Positives by PCR)	*Vibrio* Species			Total
		*V. fluvialis*	*V. parahaemolyticus*	*V. vulnificus*	
Kariega Estuary	Water (*n* = 39)	3 (7.69%)	4 (10.26%)	0 (0.00%)	7 (17.95%)
	Fish (*n* = 57)	7 (12.28%)	5 (8.77%)	1 (1.75%)	13 (22.81%)
Farm 1	Water (*n* = 73)	54 (73.97%)	4 (5.48%)	2 (2.74%)	60 (82.19%)
	Fish (*n* = 290)	109 (37.59%)	14 (11.93%)	61 (55.96%)	184 (63.45%)
Farm 2	Water (*n* = 45)	5 (11.11%)	3 (6.67%)	5 (11.11%)	13 (28.89%)
	Fish (*n* = 102)	15 (14.71%)	3 (2.94%)	5 (4.91%)	23 (22.55%)
Total	606	193 (31.85%)	33 (5.45%)	74 (12.21%)	300 (49.50%)

**Table 4:** Summary of V. fluvialis virulence genes detected from different sampling sites.

Site	*V. fluvialis* Virulence Genes			
	*vfh*	*hup*O	*stn*	*vfp*A
Farm 1 (*n* = 10)	2 (20%)	2 (20%)	2 (20%)	0 (0%)
Farm 2 (*n* = 163)	10 (6.1%)	18 (11%)	21 (12.9%)	0 (0%)
Kareiga Estuary (*n* = 20)	0 (%)	0 (0%)	3 (15%)	0 (0%)
Total (*n* = 193)	12 (6.2%)	20 (10.4%)	26 (13.5%)	0 (0%)

**Table 5:** Distribution of virulence genes in *V. fluvialis* strains.

Isolate Code	*V. fluvialis* Virulence Genes		
	*vfh*	*hupO*	*stn*
11			+
30	+	+	
36	+	+	+
57			+
58		+	+
72			+
206a		+	+
207d			+
225b		+	+
231a		+	+
241a	+	+	
241d	+	+	
241g	+	+	
241j	+	+	
252g		+	
253a		+	+
253b		+	+
253c		+	+
253d		+	+
262d			+
271a	+	+	+
271b	+	+	+
276b	+	+	
276f	+	+	
277d			+
278a	+	+	
278c			+
289	+		+
308b			+
308f			+
309a			+
330h			+
369b			+
375a			+
379a			+
Total (*n* = 35)	12	20	26
